# Harm Perceptions of E-cigarettes Among Smokers With and Without Mental Health Conditions in England: A Cross-Sectional Population Survey

**DOI:** 10.1093/ntr/ntaa020

**Published:** 2020-01-23

**Authors:** Charlie Albert Smith, Lion Shahab, Ann McNeill, Sarah E Jackson, Jamie Brown, Leonie Brose

**Affiliations:** 1 Department of Behavioural Science and Health, University College London, London, UK; 2 National Addiction Centre, King’s College London, London, UK

## Abstract

**Introduction:**

E-cigarettes (ECs) may benefit smokers with mental health conditions who are more likely to smoke, and smoke more heavily, than those without mental health conditions. This could be undermined if harm misperceptions in this group are high as is the case in the general population. This study aimed to assess EC harm perceptions relative to cigarettes as a function of mental health status and a variety of characteristics.

**Methods:**

Data were collected from 6531 current smokers in 2016/2017 in household surveys of representative samples of adults. The associations of mental health status (self-reported mental health condition and past year treatment), smoking and EC use characteristics, and characteristics relating to use of potential information sources with harm perceptions of ECs relative to cigarettes (measured by correct response “less harmful” vs. wrong responses “more harmful,” “equally harmful,” “don’t know”) were analyzed with logistic regression.

**Results:**

A similar proportion of smokers without mental health conditions (61.5%, 95% CI 60.1–62.9) and with mental health conditions (both with [61.3%, 95% confidence interval [CI] 58.7–63.8] and without past year treatment [61.5%, 95% CI 58.1–64.7]) held inaccurate EC harm perceptions (all *P* > 0.05). Being female, nonwhite, aged 25–34 compared with 16–24, from lower social grades (C2, D, and E), not having post-16 qualifications, no EC experience, a daily smoker, unmotivated to quit <1 month, non-internet user and non-broadsheet reader were all associated with more inaccurate harm perceptions (all *p* < .05).

**Conclusions:**

The majority of smokers in England have inaccurate harm perceptions of ECs regardless of mental health status.

**Implications:**

This study is the first to use a nationally representative sample in order to investigate whether smokers with and without mental health conditions differ with regard to harm perceptions of ECs. Findings show that the majority of smokers in England hold inaccurate harm perceptions of ECs, and this does not differ as a function of mental health status. A number of characteristics associated with disadvantaged groups were significantly associated with inaccurate harm perceptions. These findings highlight the need to improve awareness and understanding among disadvantaged groups regarding the relative harms of ECs compared with tobacco.

## Background

Smoking is one of the leading causes of preventable death and disability worldwide, resulting in an estimated 7 million premature deaths and 170.9 million disability-adjusted life-years lost each year.^1^ Smoking prevalence in the general population in England has declined steadily since the 1970s and in 2017 was estimated to be 14.9%^2^; however, latest data indicate prevalence remains around 50% higher among individuals with any mental health condition in the United Kingdom.^3^ Promisingly, figures show that smoking prevalence may be reducing in this population in industrialized countries such as England, although still remains substantially higher compared with the general population; falling from 44.6% in 1993 to 34.1% in 2014 in people with mental health conditions, compared with 29.1% to 19.6% within the same period for those without mental health conditions.^3^ Specifically, the available data suggest that the highest smoking prevalence is among people with illicit drug dependence and substance abuse (69%),^4^ followed by individuals with first-episode psychosis (59%),^5,6^ and individuals taking anxiolytics (41.6%) and antidepressants (33.8%).^5^ Notably, smoking prevalence can reach up to 70% among hospitalized mental health patients.^5^ However, smokers with mental health conditions are just as motivated to quit as smokers without mental health conditions,^3,7^ although those with mental health conditions experience greater dependence on smoking and long-term quit rates among this group are lower.^3,8,9^ The National Institute of Clinical and Health Excellence (NICE) have recognized that an additional approach to tobacco control is needed, and called for a tobacco harm reduction approach to be encouraged for those who are unwilling or unable to quit smoking completely which could, therefore, help with smokers with mental health conditions.^10^

E-cigarettes (ECs) are noncombustible, non-tobacco based products that mirror some behavioral aspects of smoking (eg, hand to mouth action)^11^ and appear to support smoking cessation in the general population of smokers.^12–16^ ECs have become the most popular smoking cessation aid among smokers in England,^17^ and may be able to reduce the smoking burden in the population of smokers with mental health conditions.^18^ A limited number of small randomized controlled trials (RCTs) and prospective studies have indicated that ECs increase the chances of smoking cessation among smokers with mental health conditions.^19–21^ However, it is important to note that a number of factors are likely to influence the success of ECs in this regard, including nicotine strength used,^22^ product type,^23^ and financial cost^24^; the latter may be particularly relevant for smokers from disadvantaged groups.^24^

However, smokers are only likely to convert to ECs if they believe they are less harmful than conventional cigarettes, meaning those who hold inaccurate harm perceptions may miss out on a potentially effective aid to help them quit smoking. Among a cohort of smokers and ex-smokers, one longitudinal study found that accurately perceiving ECs as less harmful than smoking predicted subsequent EC use among never users, suggesting accurate harm perceptions are important for EC initiation and subsequent use.^25^ In countries such as the United Kingdom and United States, misperceptions regarding the harms of ECs are common among the general population, and studies indicate this is getting worse.^26–29^ In addition, healthcare professionals, including those working in mental healthcare, hold many misconceptions regarding the potential harms of ECs.^30,31^ This is in spite of a consensus among prominent health organizations that ECs are significantly less harmful than cigarettes.^32–34^

Research from the United States has reported mixed findings with regard to how ECs are perceived among smokers and ex-smokers with mental health conditions. Some studies have reported that people with mental health conditions endorse positive beliefs regarding EC; both that they are less harmful than cigarettes^35,36^ and that they are helpful for smoking reduction or cessation.^37,38^ Another study found that smokers with mental health conditions were more likely to perceive both ECs and cigarettes as beneficial for weight control and for use when socializing in groups, compared with smokers without mental health conditions, although no differences were found with regard to negative physical health expectancies.^39^ Conversely, one study found smokers with current severe psychological distress reported greater perceived absolute risk of ECs and cigarettes, and were less likely to report ECs as being less harmful than cigarettes compared with smokers without severe psychological distress.^40^

It is currently unknown how ECs are perceived by smokers with mental health conditions in England and whether harm perceptions are similar, better or worse than for smokers without mental health conditions. This study, therefore, primarily aimed to compare harm perceptions of ECs relative to cigarettes between current smokers with no mental health condition, with an ever mental health condition but no past year treatment, and an ever mental health condition with past year treatment. A secondary aim was to explore associations of relative harm perceptions with demographic characteristics, smoking, and EC use characteristics. In addition, associations of relative harm perceptions with characteristics relating to use of potential information sources were explored, including internet use and newspaper readership.

## Methods

### Design and Participants

Data came from the Smoking Toolkit Study, an ongoing research program that involves monthly cross-sectional household computer-assisted interviews of ~1700 adults (aged 16+) in England using a form of random location sampling. Computer-assisted surveys are completed face-to-face and facilitated by trained interviewers from the market research company Ipsos MORI. Further details on the design of the Smoking Toolkit Study can be found elsewhere.^41^

For the present study, we used aggregated data from respondents to the survey in the period from January 2016 (the first wave to assess mental health conditions and treatment) to December 2017 (the last wave for which mental health data were available), who smoked any tobacco product at the time of the survey.

### Measures

#### Outcome Variable (Harm Perception)

To assess harm perceptions of EC, participants were asked “Compared with regular cigarettes, do you think e-cigarettes and other vaping devices are more harmful, less harmful, or equally harmful to health?” Responses included (1) less harmful than regular cigarettes, (2) equally harmful, (3) more harmful than regular cigarettes, and (4) don’t know. This was recoded as a binary variable with two levels: less harmful than regular cigarettes (the accurate response) versus all other response options (the inaccurate response).

#### Explanatory Variable (Mental Health)

Mental health was assessed by asking respondents, “Since the age of 16, which of the following, if any, has a doctor or health professional ever told you that you had.” Respondents were able to select (1) depression, (2) anxiety, (3) obsessive-compulsive disorder, (4) panic disorder/phobia, (5) post-traumatic stress disorder, (6) psychosis, (7) personality disorder, (8) attention deficit hyperactivity disorder, (9) eating disorder, (10) alcohol misuse/dependency, (11) drug use/dependency, and (12) problem gambling. Respondents who indicated not having ever been diagnosed with any of these conditions by a healthcare professional were classified as never having a mental health condition. Participants who indicated having at least one of these diagnoses were further asked “In the last 12 months, which of the following conditions, if any, have you had any treatment or taken any prescribed medication for?.” Options were filtered based on respondents’ answers (1 through 12) to the previous question. Those who indicated no treatment were classified as ever having had a mental health condition, and those who indicated having received treatment for any mental health condition in the past year were classified as having a recent mental health condition. Respondents who did not answer either question, or those who selected the options “don’t know” and/or “prefer not to say” to either question were excluded from data analysis. Therefore, three categories of mental health status were used: (1) No mental health condition, (2) Ever mental health condition but no past year treatment, and (3) Ever mental health condition with past year treatment.

### Covariates

#### Socio-Demographics

Demographic variables included sex (male/female), age (16–24, 25–34, 35–44, 45–54, 55–64, 65+ years), ethnic origin (white/nonwhite), social grade (A, B, C1/ C2, D, and E), post-16 education (no/yes) and residential region (North East, North West, and Yorkshire and the Humber collapsed as “north,” East Midlands, West Midlands and east of England collapsed as “central,” and London, South East and South West collapsed as “south”).

#### Smoking Characteristics, EC Characteristics, and Exposure to Media Channels

Additional variables included smoking frequency (daily/non-daily), high motivation to quit within the next month (yes/no), concurrent use of ECs (yes/no), and regular exposure to EC use by others (yes/no). Internet use (never/daily or less/more than daily) and regular readership of broadsheet (yes/no), mid-market (yes/no) and tabloid newspapers (yes/no) were also included in the model due to the increasingly high prevalence of news coverage regarding EC harms in these outlets.^32,42^ Broadsheet newspapers are classified as less sensationalist than tabloids, while mid-market newspapers as considered to be an intermediate.

### Analysis

The analysis plan was pre-registered on Open Science Framework (https://osf.io/zjrf5/). Data were analyzed using IBM SPSS version 24. We used Pearson’s chi-square analyses to compare descriptive characteristics between smokers in the three groups (no mental health condition, mental health condition with no past year treatment, and mental health condition with past year treatment). The relationship between the explanatory variable (mental health status) and outcome variable (harm perceptions of ECs relative to cigarettes) was analyzed using multivariable logistic regression while adjusting for covariates.

In the case of a nonsignificant association between the explanatory and outcome variable, Bayes factors (BF) were calculated using an online calculator (www.lifesci.sussex.ac.uk/home/Zoltan_Dienes/inference/Bayes.htm). A BF of ≥ 3 may be interpreted as substantial evidence for the alternative hypothesis (harm perceptions of ECs relative to regular cigarettes are worse among smokers with mental health conditions compared with smokers without mental health conditions), a BF of ≤ 1/3 may be interpreted as evidence for the null hypothesis (harm perceptions of ECs relative to regular cigarettes do not differ between smokers with and without mental health conditions), and a BF between 1/3 and 3 would suggest that the data are insensitive to detect an effect.^43,44^ Based on the mixed findings reported in the literature and our hypothesis, we decided to explore a range of expected effect sizes expressed in odds ratios, including 1.1 (small), 1.5 (medium), and 2 (large) effects of worse harm perceptions in those with mental health conditions (with and without past year treatment) versus those without.

In a sensitivity analysis, “don’t know” responses to the outcome measure were excluded to determine whether the results were influenced by the proportion of smokers who didn’t know whether ECs were more or less harmful than cigarettes.

## Results

A total of 40 831 people were surveyed between January 2016 and December 2017, of whom 7116 were current smokers of any tobacco product. Of these smokers, 6531 (91.8%) smokers provided complete data on all variables and were included in both the unadjusted and adjusted regression models (see Supplementary Table 1 for differences in characteristics between included and excluded samples). Descriptive data are presented as weighted, and unweighted data was used in the logistical regression models.

In total, 67.4% (95% CI 66.2–68.5) of the sample included in the analyses reported never having a mental health condition since the age of 16 years old, 12.4% (95% CI 11.6–13.2) reported having ever had a mental health condition without past year treatment, and 20.2% (95% CI 19.3–21.2) reported having a mental health condition and past year treatment. There were significant differences between groups with regard to age, sex, ethnicity, social grade, region of residence, post-16 education, EC use, exposure to others’ EC use, daily smoking status, motivation to quit in the next month, internet use and mid-market newspaper readership (sample characteristics are summarized in Table 1).

**Table 1.** Sociodemographic Characteristics of Sample by Mental Health Status

	No mental health condition (*n* = 4420)	Ever mental health condition without past year treatment (*n* = 797)	Ever mental health condition with past year treatment (*n* = 1314)	*p* ^a^
Demographic characteristics				
Age, % (*n*)				<.001
16–24	17.8 (802)	19.5 (157)	19.4 (264)	
25–34	21.6 (813)	24 (165)	22.2 (268)	
35–44	16.6 (645)	18 (127)	21.6 (251)	
45–54	17.8 (719)	16.6 (128)	21.4 (276)	
55–64	12.7 (645)	13.8 (130)	10.6 (176)	
65+	13.4 (796)	8.1 (90)	4.8 (79)	
Sex, % (*n*)				<.001
Men	57.9 (2526)	49.8 (389)	38.8 (514)	
Women	42.1 (1894)	50.2 (408)	61.2 (800)	
Ethnicity, %(*n*)				<.001
White	89.2 (3908)	92.9 (739)	93.2 (1218)	
Non-white	10.8 (512)	7.1 (58)	6.8 (96)	
Social grade, % (*n*)				<.001
A, B, C1	41.2 (1981)	41.7 (362)	32.7 (463)	
C2, D, E	58.8 (2439)	58.3 (435)	67.3 (851)	
Region of residence, % (*n*)				<.001
North	39.8 (1366)	34.4 (288)	36.5 (506)	
Central	29.2 (1288)	27.5 (218)	28.7 (369)	
South	41 (1766)	38 (291)	34.8 (439)	
Education level (post-16), % (*n*)				<.001
No	42.5 (1945)	36.8 (305)	45.7 (604)	
Yes	57.5 (2475)	63.2 (492)	54.3 (710)	
Smoking and EC characteristics				
Own EC use, % (*n*)				<.001
No	82.4 (3654)	77.6 (618)	76.1 (1002)	
Yes	17.6 (766)	22.4 (179)	23.9 (312)	
Exposure to others’ EC use, % (*n*)				<.01
No	73.3 (3301)	67.7 (555)	70.3 (943)	
Yes	26.7 (1119)	32.3 (242)	29.7 (371)	
Daily smoker				<.01
No	15.5 (700)	15 (125)	12 (166)	
Yes	84.5 (3720)	85 (672)	88 (1148)	
Motivation to quit smoking <1 month, % (*n*)				<.01
No	93.1 (4114)	93.5 (746)	90.7 (1189)	
Yes	6.9 (306)	6.5 (51)	9.3 (125)	
Resource use characteristics				
Internet use, % (*n*)				<.02
Never	10.8 (573)	9.3 (89)	11.1 (165)	
Daily or less	17.9 (812)	15.2 (127)	14.7 (200)	
More than daily	71.3 (3035)	75.5 (581)	74.1 (949)	
Newspaper readership				
Tabloid reader, % (*n*)				.071
No	87.9 (3989)	90.4 (756)	89.1 (1216)	
Yes	12.1 (550)	9.6 (80)	10.9 (148)	
Mid-market reader, % (*n*)				<.001
No	90.3 (3973)	91.4 (730)	93.8 (1227)	
Yes	9.7 (447)	8.6 (67)	6.2 (87)	
Broadsheet reader, % (*n*)				.126
No	90.8 (3862)	91.9 (717)	92.5 (1168)	
Yes	9.2 (558)	8.1 (80)	7.5 (146)	

EC = E-cigarette.

^a^%s are weighted. *N*s are unweighted.

A high proportion of smokers without any mental health condition held inaccurate harm perceptions (61.5%, 95% CI 60.1–62.9) and this proportion was similarly high for smokers who reported ever experiencing a mental health condition with (61.3%, 95% CI 58.7–63.8, OR 0.99, 95% CI 0.82–1.18) and without past year treatment (61.5%, 95% CI 58.1–64.7, OR 1.02, 95% CI 0.90–1.16). There were no significant group differences even after adjustment (Table 2). Being female, nonwhite, aged 25–34 (relative to age group 16–24), in lower social grade groups (C2, D, and E), not having post-16 qualifications, no experience of EC use, being a daily smoker, not being motivated to quit in the next month, not using the internet and not reading broadsheet newspapers were associated with more inaccurate harm perceptions (Table 2).

**Table 2.** Associations Between Variables and Inaccurate E-cigarette (EC) Harm Perceptions Adjusting for Covariates

	% (*n*) Inaccurate responses^a^	Adjusted *OR* (95% CI)	*p*
Mental health status			
No diagnosis	61.5 (2727)	Ref	
Ever diagnosis without past year treatment	61.3 (485)	1.03 (0.90–1.19)	.64
Ever diagnosis with past year treatment	61.5 (804)	1.07 (0.88–1.29)	.51
Age			
16–24	59.1 (713)	Ref	
25–34	65 (817)	1.28 (1.08–1.52)	<.01
35–44	60.3 (624)	1.04 (0.87–1.24)	.69
45–54	59.8 (672)	0.95 (0.80–1.14)	.57
55–64	58.3 (557)	0.83 (0.69–1.01)	.06
65+	66.4 (633)	0.99 (0.80–1.22)	.89
Gender			
Men	57.4 (1971)	Ref	
Women	66 (2045)	1.46 (1.31–1.63)	<.001
Ethnicity			
White	61 (3576)	Ref	
Non-white	65.4 (440)	1.36 (1.13–1.63)	.001
Social grade			
A, B, C1	53.8 (1539)	Ref	
C2, D, E	66.4 (2477)	1.35 (1.20–1.51)	<.001
Residential region			
North	58.6 (1282)	Ref	
Central	63.4 (1192)	1.11 (0.97–1.27)	.12
South	62.2 (1542)	1.08 (0.95–1.23)	.23
Education level (post-16)			
No	66 (1900)	Ref	
Yes	58 (2116)	0.87 (0.77–0.97)	<.02
Own EC use			
No	67.4 (3552)	Ref	
Yes	36.9 (464)	0.30 (0.26–0.35)	<.001
Exposure to others’ EC use			
No	64 (3071)	Ref	
Yes	55.7 (945)	0.90 (0.80–1.02)	.09
Daily smoker			
No	52.9 (526)	Ref	
Yes	62.9 (3490)	1.30 (1.12–1.50)	.001
Motivation to quit <1 month			
No	62.1 (3760)	Ref	
Yes	52.7 (256)	0.79 (0.64–0.96)	<.02
Internet use			
Never	72.8 (605)	Ref	
Daily or less	63.7 (718)	0.73 (0.59–0.90)	<.01
More than daily	59.2 (2693)	0.66 (0.54–0.80)	<.001
Tabloid reader			
No	61 (3501)	Ref	
Yes	64.5 (515)	1.10 (0.93–1.30)	.27
Mid-market reader			
No	61.5 (3652)	Ref	
Yes	60.9 (364)	1.00 (0.83–1.20)	.96
Broadsheet reader			
No	62.6 (3739)	Ref	
Yes	48.7 (277)	0.68 (0.57–0.82)	<.001

^a^%s are weighted. *N*s, odds ratios (*OR*s), and 95% confidence intervals (CIs) are unweighted.

BF indicated that data were insensitive to rule out small (BF = 0.93) or medium-sized effects (BF = 0.43) but did provide substantial evidence for the null hypothesis for there being no large (OR 2.0) differences in harm perceptions between those without and with a mental health conditions without past year treatment (BF = 0.26). BF indicated that data were insensitive to rule out small (BF = 1.01), medium (BF = 0.61) and large-sized effects (BF = 0.4) for there being differences in harm perceptions between those without and with a mental health conditions with past year treatment.

Finally, a sensitivity analysis that excluded “don’t know” responses (adjusted *n* = 810) to the outcome variable (harm perception) produced similar results to those of the adjusted regression model with regard to mental health. Specifically, a similar proportion of smokers without mental health conditions (56.2%; 95% CI 54.6–57.7) and with mental health conditions (both with [56.5%; 95% CI 53.7–59.3] and without past year treatment [56.9%; 95% CI 53.3–60.4]) held inaccurate EC harm perceptions (all *p* > .05).

## Discussion

To our knowledge, this is the first study to use nationally representative data to explore smokers’ harm perceptions of ECs relative to cigarettes as a function of mental health status in England. The findings show that harm perceptions of ECs relative to regular cigarettes among current smokers in England are generally inaccurate. Specifically, the majority (61.4%) of smokers have inaccurate harm perceptions of ECs relative to cigarettes. There was no evidence of an association between mental health status and harm perceptions of ECs; that is, having ever had a mental health condition since the age of 16, and receiving past year treatment for a mental health condition, were not associated with smokers’ harm perceptions of ECs relative to cigarettes. This finding persisted following adjustment for potential confounders.

It is well-established that perceptions regarding harms of ECs relative to cigarettes are generally inaccurate among past year smokers in the United Kingdom; one study reported that harm perceptions of ECs worsened between 2012 (33.4%) and 2014 (39.6%),^25^ and a recent study using UK nationally representative data from an online survey in September 2017 reported 42.7% had inaccurate harm perceptions of ECs.^29^ Among current smokers, Wilson et al. (2019) reported that 45.4% had inaccurate harm perceptions of ECs. According to findings from the present study, 61.4% of current smokers had inaccurate harm perceptions regarding ECs, demonstrating that harm perceptions among smokers in England may be worsening over time. Given that accurate harm perceptions of ECs have been associated with subsequent use,^25^ it is feasible that this may deter smokers in England from trying ECs as an alternative to smoking.

We found that harm perceptions of ECs relative to cigarettes did not differ as a function of mental health status. This suggests that smokers with and without mental health conditions in the present study may be exposed to similar negative influences, or alternatively are not exposed to accurate resources and messages; particularly as a similarly large proportion of smokers in all groups held inaccurate harm perceptions. Indeed, similarities in negative expectancies (including physical and future health concerns) among smokers with and without mental health conditions has been reported in a US study by Miller et al.^39^ However, Miller et al.^39^ found smokers had fewer health concerns regarding ECs in comparison with cigarettes. Moreover, our findings contradict those from one US study which found smokers with severe psychological distress had greater absolute risk perceptions of ECs and cigarettes, and were less likely to believe that ECs are less harmful than cigarettes compared with smokers without severe psychological distress.^40^ Conversely, another study reported that a majority of current and ex-smokers with mental health conditions agreed that ECs are less harmful than cigarettes.^36^ However, it is important to note that the methodologies of these aforementioned studies differ from the present study as they used data from non-representative US samples. Moreover, differences exist with regard to the inclusion of covariates and measures used to categorize mental health status, which may partially account for the differences found.

Additional findings of the present study include the significant association between EC use and correctly believing ECs are less harmful than cigarettes, which confirms findings from previous studies.^25,28,29^ Moreover, being motivated to quit in the next month and smoking less than daily were also associated with accurately perceiving ECs as less harmful than cigarettes, which is what may be expected as these smokers are more likely to be receiving smoking cessation/harm reduction advice and be preparing for a quit attempt. We found that being from non-white ethnicity, lower Socioeconomic status, and not having higher-level (post-16) qualifications were significantly associated with more inaccurate harm perceptions of ECs, which has been reported elsewhere^45^ and may be due to low-level awareness of ECs in these groups.^46^ Finally, this study found that smokers who use the internet at least daily were more likely to identify ECs as safer alternatives to cigarettes, as were smokers who were regular readers of broadsheet newspapers. These are novel findings and may imply that access to certain resources may improve or impair smokers’ harm perceptions regarding ECs. This is in agreement with research showing that exposure to negative EC news headlines increased beliefs about EC harms compared with exposure to positive headlines.^47^

A strength of this study is that it is the first to provide insight into smokers’ harm perceptions of ECs in relation to mental health status in England. Moreover, data were drawn from a nationally representative sample of current smokers in England, although this also means findings may not be generalizable to the rest of the UK, as well as other countries or clinical populations. Indeed, the Smoking Toolkit Study is a household survey, and the sample drawn from the community and so would not necessarily include those with more severe illnesses where smoking prevalence is higher (ie, inpatient facilities).^48^ Similarly, there was not enough data to compare harm perceptions between mental health conditions, which is a major limitation of the present study as the mental health population is heterogenous. Moreover, as data are cross-sectional, conclusions cannot be made regarding causality. In addition, due to the nature of Smoking Toolkit Study, mental health status was self-reported, and so this increases the risk for potential bias. Finally, the data from the present study only captured harm perceptions held among smokers between January 2016 and December 2017. Changes in harm perceptions regarding ECs among the British population have been reported over past years, and are likely to continue changing following the emergence of new research, public health guidance and recommendations, and media coverage.^25,29,49^ Therefore, findings should be interpreted in the time context in which they were collected.

To conclude, inaccurate harm perceptions of ECs relative to cigarettes remain disproportionately high among smokers with and without mental health conditions in England but do not differ as a function of mental health status. Future research should continue to monitor harm perceptions regarding ECs among smokers, as well as explore how to efficiently disseminate public health messages inferred from the growing body of scientific evidence supporting ECs as being substantially less harmful than cigarettes. This is particularly important for vulnerable populations such as smokers with mental health conditions who experience higher dependence on smoking and so may benefit more from ECs as a harm reduction tool.

## Supplementary Material

A Contributorship Form detailing each author’s specific involvement with this content, as well as any supplementary data, are available online at https://academic.oup.com/ntr.

ntaa020_suppl_Supplementary_File

ntaa020_suppl_Supplementary_Taxonomy-Form
