# Supplementary material for: Harm Perceptions of E-cigarettes Among Smokers With and Without Mental Health Conditions in England: A Cross-Sectional Population Survey
Source: Nicotine Tob Res. 2020 Jan 23;23(3):511–7. doi: 10.1093/ntr/ntaa020 (PMC7885771; doi:10.1093/ntr/ntaa020)
Supplement: ntaa020_suppl_Supplementary_File [file ntaa020_suppl_supplementary_file.docx]

**Supplementary files**

**Table S1: Sociodemographic characteristics of participants included and excluded from unadjusted and adjusted regression models.**

|  | **Excluded from adjusted regression model (n=585)** | **Included in adjusted regression model (n=6,531)** | **P value** |
| --- | --- | --- | --- |
| **Demographic characteristics** | | | |
| **Age, %(n)** | | | .78 |
| 16-24 | 16.8 (97) | 18.4 (1,223) |  |
| 25-34 | 22.2 (110) | 22 (1,246) |  |
| 35-44 | 18.3 (97) | 17.8 (1,023) |  |
| 45-54 | 20.4 (113) | 18.4 (1,123) |  |
| 55-64 | 12.3 (87) | 12.4 (951) |  |
| 65+ | 10 (81) | 11 (965) |  |
| **Sex, % (n)** | | | .31 |
| Men | 50.9 (295) | 53 (3,429) |  |
| Women | 49.1 (288) | 47 (3,102) |  |
| **Ethnicity, % (n)** | | | .37 |
| White | 89.3 (488) | 90.5 (5,865) |  |
| Non-white | 10.7 (60) | 9.5 (666) |  |
| **Social grade, % (n)** | | | .03 |
| A, B, C1 | 35.1 (226) | 39.6 (2,806) |  |
| C2, D, E | 64.9 (359) | 60.4 (3,725) |  |
| **Region of residence, % (n)** | | | .64 |
| North | 31.5 (190) | 31.8 (2,160) |  |
| Central | 30.6 (177) | 28.9 (1,875) |  |
| South | 37.9 (215) | 39.4 (2,496) |  |
| **Education level (post 16), % (n)** | | | .25 |
| No | 44.9 (251) | 42.4(2,854) |  |
| Yes | 55.1 (287) | 57.6 (3,677) |  |
| **Mental health status** | | | .15 |
| No mental health condition | 72.7 (50) | 67.4 (4,420) |  |
| Mental health condition without past year treatment | 4.5 (4) | 12.4 (797) |  |
| Mental health condition with past year treatment | 22.7 (15) | 20.2 (1,314) |  |
| **Smoking characteristics** | | | |
| **Own EC use, % (n)** |  |  | .42 |
| No | 81.9 (485) | 80.5 (5,274) |  |
| Yes | 18.1 (100) | 19.5 (1,257) |  |
| **Exposure to others’ EC use, % (n)** | | | .15 |
| No | 74.7 (446) | 72 (4,799) |  |
| Yes | 25.3 (139) | 28 (1,732) |  |
| **Daily smoker** | | | <.01 |
| No | 18.8 (108) | 14.7 (991) |  |
| Yes | 81.2 (477) | 85.3 (5,540) |  |
| **Motivation to quit <1 month, % (n)** | | |  |
| No | 91.4 (537) | 92.7 (6,049) | .26 |
| Yes | 8.6 (48) | 7.3 (482) |  |
| **Resource use characteristics** | | | |
| **Internet use, % (n)** | | | .14 |
| Never | 13.3 (92) | 10.7 (827) |  |
| Daily or less | 16.3 (105) | 16.9 (1,139) |  |
| More than daily | 70.4 (388) | 72.4 (4,565) |  |
| **Newspaper readership** | | | |
| **Tabloid reader, % (n)** | | | .56 |
| No | 87.7 (508) | 88.5 (5,747) |  |
| Yes | 12.3 (75) | 11.5 (784) |  |
| **Mid-market reader, % (n)** | | | .81 |
| No | 91.4 (529) | 91.1 (5,930) |  |
| Yes | 8.6 (54) | 8.9 (601) |  |
| **Broadsheet reader, % (n)** | | | .19 |
| No | 92.9 (541) | 91.3 (5,950) |  |
| Yes | 7.1 (42) | 8.7 (581) |  |

^*%s are weighted. Ns are unweighted.^
